# Acute heart failure with and without acute coronary syndrome: clinical correlates and prognostic impact (From the HEARTS registry)

**DOI:** 10.1186/s12872-016-0267-6

**Published:** 2016-05-20

**Authors:** Hussam AlFaleh, Abdelfatah A. Elasfar, Anhar Ullah, Khalid F. AlHabib, Ahmad Hersi, Layth Mimish, Ali Almasood, Saleh Al Ghamdi, Abdullah Ghabashi, Asif Malik, Gamal A. Hussein, Mushabab Al-Murayeh, Ahmed Abuosa, Waleed Al Habeeb, Tarek S. Kashour

**Affiliations:** Department of Cardiac Sciences, College of Medicine, King Saud University, Riyadh, Saudi Arabia; King Salman Heart Center, King Fahd Medical City, Riyadh, Saudi Arabia; King Abdulaziz University Hospital, King Abdulaziz University, Jeddah, Saudi Arabia; Prince Sultan Cardiac Center, Riyadh, Saudi Arabia; Madina Cardiac Center, Al Madina Al Monaoarah, Saudi Arabia; Prince Sultan Cardiac Center, Hafouf, Saudi Arabia; King Fahad General Hospital, Jeddah, Saudi Arabia; North West Armed Forces Hospital, Tabuk, Saudi Arabia; Armed Forces Hospital Southern Region, Khamis Mushayt, Saudi Arabia; National Guard Hospital, Jeddah, Saudi Arabia; Cardiac Sciences, King Khalid University Hospital, College of Medicine, King Saud University, P.O. Box 7805, Riyadh, 11472 Saudi Arabia

**Keywords:** Heart failure complications/mortality/physiopathology, Acute coronary syndrome complications/mortality/physiopathology, Hospital Mortality, Saudi Arabia/epidemiology, Prospective Studies

## Abstract

**Background:**

Little is know about the outcomes of acute heart failure (AHF) with acute coronary syndrome (ACS-AHF), compared to those without ACS (NACS-AHF).

**Methods:**

We conducted a prospective registry of AHF patients involving 18 hospitals in Saudi Arabia between October 2009 and December 2010. In this sub-study, we compared the clinical correlates, management and hospital course, as well as short, and long-term outcomes between AHF patients with and without ACS.

**Results:**

Of the 2609 AHF patients enrolled, 27.8 % presented with ACS. Compared to NACS-AHF patients, ACS-AHF patients were more likely to be old males (Mean age = 62.7 vs. 60.8 years, *p* = 0.003, and 73.8 % vs. 62.7 %, *p* < 0.001, respectively), and to present with De-novo heart failure (56.6 % vs. 28.1 %, *p* < 0.001). Additionally they were more likely to have history of ischemic heart disease, diabetes, dyslipidemia, and less likely to have chronic kidney disease (*p* < 0.001 for all comparisons). The prevalence of severe LV systolic dysfunction (EF < 30 %) was higher in ACS-AHF patients. During hospital stay, ACS-AHF patients were more likely to develop shock (*p* < 0.001), recurrent heart failure (*p* = 0.02) and needed more mechanical ventilation (*p* < 0.001). β blockers and Angiotensin Converting Enzyme inhibitors were used more often in ACS-AHF patients (*p* = 0.001 and, *p* = 0.004 respectively). ACS- AHF patients underwent more coronary angiography and had higher prevalence of multi-vessel coronary artery disease (*p* < 0.001 for all comparisons). The unadjusted hospital and one-month mortality were higher in ACS-AHF patients (OR = 1.6 (1.2–2.2), *p* = 0.003 and 1.4 (1.0–1.9), *p* = 0.026 respectively). A significant interaction existed between the level of left ventricular ejection fraction and ACS-AHF status. After adjustment, ACS-AHF status was only significantly associated with hospital mortality (OR = 1.6 (1.1–2.4), *p* = 0.019). The three-years survival following hospital discharge was not different between the two groups.

**Conclusion:**

AHF patients presenting with ACS had worse hospital prognosis, and an equivalent long-term survival compared to AHF patients without ACS. These findings underscore the importance of timely recognition and management of AHF patients with concomitant ACS given their distinct presentation and underlying pathophysiology compared to other AHF patients.

## Background

Heart failure (HF) continues to be a significant cause of morbidity and mortality worldwide [1–3], and a prior history of coronary artery disease is present in over half of the acute HF (AHF) patients admitted to the hospital [4–8]. Data from acute coronary syndrome (ACS) registries indicate that ACS complicated by HF (ACS-AHF) leads to a several-fold increase in hospital mortality compared to those without AHF [9–11]. Additionally, the adverse effects of ACS-AHF appear to extend beyond hospital discharge and up to one year following the index event [11, 12]. Moreover, a substantial proportion of hospitalized ACS patients develop AHF during their hospital course, and carries worse prognosis than those who present initially with ACS and AHF. Despite the high-risk status of ACS-AHF patients-, these patients are undertreated compared to ACS patients without AHF [13]. Although the clinical characteristics, therapies, and outcomes of ACS-AHF are well described in the context of ACS registries, i.e. in patients who present initially and overtly with ACS, little is known about the outcomes of these patients compared to the outcomes of AHF patients without concomitant ACS (NACS-AHF). AHF represents a syndrome that has a heterogeneous pathophysiology with variable outcomes. Thus, it is of interest to compare a group with unique pathophysiology and therapeutic targets such as ACS-AHF to NACS-AHF patients. Accordingly, we determined the prevalence, clinical correlates, and hospital therapies of ACS-AHF in a large contemporary HF registry. Additionally, we explored the impact of ACS presentation on hospital outcomes and on the short- and long-term mortality of patients hospitalized for AHF.

## Methods

The design and rational of the HEARTS registry were described previously [2, 14]. Briefly, HEARTS is the first prospective HF registry in Saudi Arabia and the Arab Middle East. It enrolled 2609 consecutive patients who were admitted to the hospital with a primary diagnosis of AHF. Patients younger than 18 years of age who were unable to provide consent were excluded from the study. The registry included data from 18 hospitals in different regions of Saudi Arabia between October 2009 and December 2010, with follow-up until January 2013. Six of the 18 hospitals participating in this study did not have cardiac catheterization laboratories or cardiac surgery facilities. The diagnosis of AHF was made according to the European Society of Cardiology guidelines for the diagnosis and management of acute and chronic HF [15]. For this analysis, we stratified the enrolled AHF patient population into two groups: those who had evidence of ACS concomitant with AHF at hospital presentation (ACS-AHF) vs. those without ACS (NACS-AHF). The ACS group comprised patients with ST elevation myocardial infarction (STEMI) and patients with non-ST acute coronary syndrome (NSTACS). The treating physician determined which patients had ACS based on the presenting symptoms and clinical context, on electrocardiography (ECG) findings, as well as on cardiac biomarker levels. The definitions for STEMI and NSTACS were based on the American College of Cardiology clinical data standards [16, 17]. Troponin assays were performed at initial emergency department assessment and were performed at the physicians’ discretion. The assay cut-off values were determined by each participating hospital biochemistry lab according to their values for what constituted a positive troponin level. Data for 30-day, 1-year, 2-year, and 3-year all-cause mortality were obtained from each hospital by a telephone inquiry. We compared the clinical characteristics, hospital management, and adverse outcomes, as well as short-term and long-term all-cause mortality, following the index admission between the two groups. The study was approved by the institutional review board at each hospital and complied with the tenets of the Helsinki Declaration. The study was granted a waiver for consent because the study carried no more than minimal risk for the patients, the waiver do not affect adversely the rights and welfare of recruited patients and data management of the study carried no more than minimal risk to privacy of the patients and maintained high standards for confidentiality. Furthermore, verbal consent was obtained from recruited patients “out of courtesy” and to establish rapport with them.

### Statistical analysis

Categorical data were summarized as absolute numbers and percentages. Numeric data were summarized as means and standard deviations (SD) or as medians and interquartile ranges. Comparisons between different groups were performed using the chi-square test or Fisher’s exact for categorical variables and independent sample t-test or the Mann-Whitney U test for continuous variables. The adjusted odds ratios were estimated using multiple logistic regression. Model adjustment was performed using the following variables: age, sex, history of HF, ischemic heart disease (IHD), history of percutaneous coronary intervention (PCI), history of coronary artery bypass surgery (CABG), diabetes mellitus (DM), hypertension, anemia, body mass index, estimated glomerular filtration rate (eGFR) as calculated by the chronic kidney disease epidemiology collaboration equation (CKD-EPI) [18], systolic blood pressure, diastolic blood pressure, heart rate, AHF type (de novo and acute on chronic HF), and LV systolic dysfunction. A multiple logistic regression model with stepwise selection and backward elimination was used to identify predictors of 3-year mortality, and a 5 % significance level was used to remain in the model. Logistic regression with an interaction term was used to test the statistical significance of interactions between selected study groups, and we estimated the strength of the associations of these groups using odds ratios with 95 % confidence intervals. Kaplan-Meier analysis was used to plot the cumulative survival, and differences between groups were assessed by the log-rank test. All analyses were performed using SAS/STAT software, version 9.2 (SAS Institute Inc., Cary, NC, USA) and R (Foundation for Statistical Computing, Vienna, Austria). A 2-sided *p*-value < 0.05 was considered statistically significant.

## Results

### Clinical characteristics

Of the 2609 patients with AHF who were included in this study, 725 (27.8 %) patients had concomitant ACS (38.1 % STEMI and 61.9 % NSTACS). Compared to NACS-AHF patients, patients with ACS-AHF were on average two years older (*p* = 0.003) and were more likely to be male (*p* < 0.001) (Table 1). Risk factors for atherosclerosis such as DM, hyperlipidemia, and smoking were significantly more common in ACS-AHF patients. Moreover, established vascular disease, such as IHD and peripheral arterial disease, was more frequent in ACS-AHF patients. On the other hand, 72.2 % of NACS-AHF patients had a past history of HF compared to 43.5 % of ACS-AHF patients (*p* < 0.001), and NACS-AHF patients more often had other comorbidities such as a past history of rheumatic heart disease, atrial fibrillation (AF), anemia, CKD, chronic liver disease, thyroid disease, and chronic lung disease.

**Table 1:** Baseline characteristics of acute heart failure patients with and without acute coronary syndrome

	Overall *n* = 2609	ACS-AHF *n* = 725 (27.79 %)	NACS-ACS *n* = 1884 (72.21 %)	*P* value
Demographics				
Age, years; mean ± SD	61.34 ± 15	62.74 ± 13.1	60.80 ± 15.6	0.003
Male, n (%)	1717 (65.81)	535 (73.79)	1182 (62.74)	<0.001
Saudi nationality, n (%)	2230 (85.47)	546 (75.31)	1684 (89.38)	<0.001
Medical history				
Heart failure, n (%)	1670 (64.2)	315 (43.5)	1355 (72.2)	<0.001
Ischemic heart disease, n (%)	1376 (53.3)	433 (60.1)	943 (50.6)	<0.001
PCI, n (%)	340 (13.1)	109 (15.0)	231 (12.3)	0.064
CABG, n (%)	261 (10.0)	52 (7.2)	209 (11.1)	0.003
Rheumatic heart disease, n (%)	183 (7.1)	4 (0.5)	179 (9.6)	<0.001
Atrial fibrillation, n (%)	408 (15.7)	37 (5.1)	371 (19.8)	<0.001
ICD, n (%)	229 (8.8)	31 (4.3)	198 (10.5)	<0.001
CRT, n (%)	85 (3.3)	7 (0.8)	78 (4.1)	<0.001
Stroke/TIA, n (%)	252 (9.7)	67 (9.2)	185 (9.8)	0.637
PAD, n (%)	99 (3.8)	37 (5.1)	62 (3.3)	0.034
Anemia, n (%)	1166 (44.9)	301 (41.7)	865 (46.2)	0.043
Chronic lung disease, n (%)	185 (7.1)	37 (5.1)	148 (7.9)	0.013
Chronic kidney disease, n (%)	771 (29.7)	163 (22.5)	608 (32.4)	<0.001
Liver disease, n (%)	91 (3.5)	9 (1.2)	82 (4.4)	<0.001
Thyroid disorder, n (%)	172 (6.8)	28 (4.0)	144 (7.9)	<0.001
Risk factors for atherosclerosis				
Smoking, n (%)	467 (17.9)	182 (25.1)	285 (15.1)	<0.001
Hypertension, n (%)	1831 (70.6)	506 (70.8)	1325 (70.5)	0.914
Dyslipidemia, n (%)	894 (36.4)	289 (42.7)	605 (34.0)	<0.001
Diabetes mellitus, n (%)	1668 (64.1)	518 (72.0)	1150 (61.1)	<0.001
Clinical parameters on presentation				
Acute de novo HF, n (%)	939 (36.0)	410 (56.5)	529 (28.1)	<0.001
Acute on chronic HF, n (%)	1670 (64.0)	315 (43.4)	1355 (71.9)	
BMI, kg/m^2^; mean ± SD*	29.16 ± 6.7	28.12 ± 5.4	29.55 ± 7.1	<0.001
SBP, mean ± SD	128.7 ± 31.3	126.6 ± 29.8	129.5 ± 31.9	0.038
DBP, mean ± SD	74.10 ± 17.9	73.67 ± 17.1	74.26 ± 18.2	0.454
HR, mean ± SD	88.8 ± 21.0	89.7 ± 19.1	88.5 ± 21.7	0.185
Investigations				
Electrocardiography				
Atrial fibrillation/flutter, n (%)	449 (17.2)	52 (7.2)	397 (21.1)	<0.001
QRS ≥120 msec, n (%)	389 (14.9)	68 (9.4)	321 (17.1)	<0.001
LBBB, n (%)	305 (11.7)	62 (8.5)	243 (12.9)	0.002
Biochemical parameters				
Sodium, mmol/L; mean ± SD	135.1 ± 5.3	135.0 ± 5.4	135.2 ± 5.3	0.322
Potassium, mmol/L; mean ± SD	4.3 ± 0.7	4.2 ± 0.7	4.3 ± 0.7	0.644
Urea, μmol/L; mean ± SD	11.87 ± 9.1	11.11 ± 8.4	12.16 ± 9.3	0.008
Creatinine, μmol/L; median (IQR]	109.0 (70.0)	109.0 (62.0)	109.0 (73.0)	0.923
eGFR, ml/min/1.73 m^2^; median (IQR)	57.28 (43.5)	56.32 (41.9)	57.82 (44.1)	0.444
Hemoglobin, g/dL; mean ± SD	12.43 ± 2.2	12.76 ± 2.3	12.31 ± 2.2	<0.001
RBS, mmol/L; mean ± SD	10.01 ± 5.7	11.43 ± 6.2	9.45 ± 5.4	<0.001
NT-Pro-BNP, pmol/L; n (%)	435 (16.7)	101 (13.9)	334 (17.7)	0.019
NT-Pro-BNP, pmol/L; median (IQR)	675.0 (668)	999.0 (1679)	631.5 (684)	<0.001
Positive troponin, n (%)	867 (37.4)	693 (98.6)	174 (10.8)	<0.001
Echocardiography				
Left ventricular EF >55 %, n (%)	341 (13.7)	50 (7.2)	291 (16.2)	<0.001
Left ventricular EF 40–54.9 %	334 (13.4)	113 (16.2)	221 (12.3)	<0.001
Left ventricular EF 30–9.9 %	632 (25.3)	258 (37.1)	374 (20.8)	<0.001
Left ventricular EF <30 %	1187 (47.6)	275 (39.5)	912 (50.7)	<0.001

*Abbreviations*: *ACS-AHF* acute coronary syndrome with acute heart failure, *ACS-NASC* no acute coronary syndrome with acute heart failure, *BMI* body mass index, *BNP* brain natriuretic peptide, *CABG* coronary artery bypass surgery, *CRT* cardiac resynchronization therapy, *DBP* diastolic blood pressure, *EF* ejection fraction, *eGFR* estimated glomerular filtration rate, *HR* heart rate, *ICD* internal cardiac defibrillator, *LBBB* left bundle branch block, *PAD* peripheral artery disease, *PCI* percutaneous coronary intervention, *RBS* random blood sugar, *SD* standard deviation, *SBP* systolic blood pressure, *TIA* transient ischemic attack

Several differences were noted with respect to clinical presentation. A significant proportion of ACS-AHF patients presented with de novo HF (56.6 %), while 71.9 % of NACS-AHF patients presented with acute on chronic HF (*p* < 0.001). NACS-AHF patients were more likely to present with AF (21.1 % vs. 7.2 %, *p* < 0.001) and to have a QRS duration ≥120 ms. Significant differences between the two groups were noted for several biochemical parameters (Table 1). Notably, ACS-AHF patients had significantly higher mean random blood sugar (RBS), with the mean value (11.43 ± 6.2 mmol/l) being in the hyperglycemia range; on the other hand, NACS-AHF patients had lower hemoglobin levels (*p* < 0.001 for both comparisons). Approximately 10 % of the NACS-AHF group were positive for troponin I. Serum NT-ProBNP testing was only performed in 16.7 % of the total cohort, and its concentration in the ACS-AHF group was more than double that in the NACS-AHF group (*p* < 0.001). Patients with NACS-AHF were more likely to have severe left ventricular (LV) systolic dysfunction (ejection fraction (EF) <30 %) on echocardiography compared to ACS-AHF patients. In contrast, ACS-AHF patients were more likely to have mild and moderate LV systolic dysfunction (Table 1).

### Hospital procedures and medical therapy

The use of intra-aortic balloon pumps (IABP) and mechanical ventilation was higher in ACS-AHF patients (Table 2). Coronary angiography (CAG) was performed in 29.3 % of the total cohort, and its performance was more than double in ACS-AHF compared to NACS-AHF patients. Single- or multi-vessel coronary disease was documented more frequently in ACS-AHF patients, while normal or non-significant coronary artery disease was more frequent in NACS-AHF patients. The use of intravenous nitroglycerine and dopamine was greater in ACS-AHF patients. Upon discharge, patients with ACS-AHF were more likely to be prescribed aspirin, statins, β-blockers, and angiotensin-converting enzyme (ACE) inhibitors. Conversely, the use of diuretics (furosemide and metolazone), aldosterone antagonists (AAs), hydralazine, and oral anticoagulants was higher in patients with NACS-AHF (Table 2).

**Table 2:** Hospital procedures, therapies, and discharge medications in acute heart failure patients with or without ACS

	Overall *n* = 2609	ACS-AHF *n* = 725 (27.79 %)	NACS-ACS *n* = 1884 (72.21)	*P* value
Hospital procedures and therapies				
IV nitroglycerine, n (%)	725 (27.8)	397 (54.8)	328 (17.4)	<0.001
Dopamine, n (%)	468 (17.9)	156 (21.5)	312 (16.6)	0.003
Invasive ventilation (%)	289 (11.1)	106 (14.6)	183 (9.7)	<0.001
IABP, n (%)	86 (3.3)	57 (7.9)	29 (1.5)	<0.001
Cardiac pacing, n (%)	36 (1.38)	18 (2.48)	18 (0.96)	0.003
Hospital ICD, n (%)	150 (5.75)	32 (4.41)	118 (6.26)	0.069
Hospital CRT, n (%)	68 (2.61)	9 (1.24)	59 (3.13)	0.007
CAG performed, n (%)	764 (29.3)	338 (46.6)	426 (22.6)	<0.001
Normal coronaries, n (%)	183 (24.0)	9 (2.7)	174 (40.9)	<0.001
Non-significant CAD	82 (10.7)	14 (4.1)	68 (16.0)	<0.001
Single-vessel CAD, n (%)	105 (13.7)	56 (16.6)	49 (11.5)	0.043
Double-vessel CAD, n (%)	116 (15.2)	77 (22.8)	39 (9.2)	<0.001
LM or three-vessel CAD, n (%)	263 (34.4)	179 (53.0)	84 (19.7)	<0.001
Discharge medications				
Aspirin, n (%)	1989 (76.2)	633 (87.3)	1356 (72.0)	<0.001
Warfarin, n (%)	484 (18.5)	56 (7.7)	428 (22.7)	<0.001
Statin, n (%)	1813 (69.5)	605 (83.4)	1208 (64.1)	<0.001
Lasix, n (%)	2048 (78.5)	546 (75.3)	1502 (79.7)	0.014
Metolazone, n (%)	115 (4.4)	17 (2.3)	98 (5.2)	0.001
Hydralazine, n (%)	300 (11.5)	61 (8.4)	239 (12.7)	0.002
Long-acting nitrates, n (%)	252 (9.7)	103 (14.2)	149 (7.9)	<0.001
Amiodarone, n (%)	75 (2.9)	14 (1.9)	61 (3.2)	0.074
Βeta-blockers, n (%)	2180 (83.0)	635 (87.6)	1545 (82.0)	<0.001
ACEI, n (%)	1554 (59.6)	463 (63.9)	1091 (57.9)	0.006
ARBI, n (%)	388 (14.9)	95 (13.1)	293 (15.5)	0.115
AA, n (%)	999 (38.4)	191 (26.3)	808 (42.9)	<0.001

*Abbreviations*: *AA* aldosterone antagonist, *ACEI* angiotensin-converting enzyme inhibitor, *ACS-AHF* acute coronary syndrome with acute heart failure, *ACS-NASC* no acute coronary syndrome with acute heart failure, *ARBI* angiotensin receptor blocker, *CAG* coronary angiography, *CRT* cardiac resynchronization therapy, *IABP* intra-aortic balloon pump, *ICD* internal cardiac defibrillator, *IV* intravenous, *LM* left main

### Hospital course

ACS-AHF patients were more likely to suffer from shock (cardiogenic, non-cardiogenic, or both), although there was no significant difference in the rate of cardiogenic shock between the two groups (*p* = 0.132) (Table 3). Additionally, the rate of recurrent HF, sepsis, and stroke or transient ischemic attack (TIA) was higher in ACS-AHF patients (Table 3). Malignant ventricular arrhythmias requiring therapy and pacing rates for brady-arrhythmias were encountered more frequently in ACS-AHF patients, but AF requiring therapy was more frequent in NACS-AHF patients.

**Table 3:** Clinical outcomes in acute heart failure patients with or without acute coronary syndrome

	Overall *n* = 2609	ACS-AHF *n* = 725 (27.79 %)	NACS-ACS *n* = 1884 (72.21)	*P* value
Hospital course and outcomes				
Recurrent CHF, n (%)	816 (31.3)	251 (34.6)	565 (30.0)	0.022
Overall shock, n (%)	228 (8.7)	94 (13.0)	134 (7.1)	<0.001
Cardiogenic, n (%)	169 (74.1)	73 (77.7)	96 (71.6)	0.132
Non-cardiogenic, n (%)	22 (9.6)	11 (11.7)	11 (8.2)	
Mixed, n (%)	37 (16.2)	10 (10.6)	27 (20.1)	
VT/VF requiring therapy, n (%)	110 (4.2)	45 (6.2)	65 (3.4)	0.002
AF requiring therapy, n (%)	156 (6.0)	25 (3.4)	131 (6.9)	<0.001
Sepsis, n (%)	196 (7.5)	68 (9.4)	128 (6.8)	0.025
Major bleeding, n (%)	38 (1.5)	14 (1.9)	24 (1.4)	0.209
Stroke/TIA, n (%)	48 (1.8)	23 (3.2)	25 (1.3)	0.002
Hospital stay, days; mean ± SD	12.3 ± 14.6	13.1 ± 15.38	12.0 ± 14.3	0.083
Hospital stay, days; median (IQR)	8.0 (9.0)	8.0 (10.0)	8.0 (9.0)	0.111
All-cause mortality				
Hospital, n (%)	170 (6.5)	64 (8.8)	106 (5.6)	0.003
1 month, n (%)	212 (8.1)	73 (10.1)	139 (7.4)	0.044
1 year, n (%)	509 (19.5)	147 (20.3)	362 (19.2)	0.568
2 years, n (%)	615 (23.6)	171 (23.6)	444 (23.6)	0.680
3 years, n (%)	635 (24.3)	175 (24.1)	460 (24.4)	0.671

*Abbreviations*: *ACS-AHF* acute coronary syndrome with acute heart failure, *ACS-NASC* no acute coronary syndrome with acute heart failure, *VT* ventricular tachycardia, *VF* ventricular fibrillation, *AF* atrial fibrillation, *TIA* transient ischemic attacks

### Mortality

Overall hospital, 30-day, 1-year, 2-year and 3-year cumulative mortality data are presented in Table 3. The Crude hospital and 30-day mortality rates were significantly higher in ACS-AHF patients (8.8 % vs. 5.6 %, *p* = 0.003 and 10.1 % vs. 7.4 %, *p* = 0.04, respectively), while there were no differences in the 1-, 2-, or 3-year cumulative mortality rates between the two groups.

The unadjusted hospital and 30-day mortality rates were significantly higher in ACS-AHF patients (OR = 1.6 (95 % CI, 1.2–2.2), *p* = 0.003 and 1.40 (95 % CI, 1.0–1.9), *p* = 0.026, respectively) (Table 4). After adjustment for important demographic characteristics, past vascular history, important comorbidity risk factors for atherosclerosis, renal function, and LV dysfunction, ACS-AHF was only predictive of hospital mortality (OR = 1.61 (95 % CI, 1.1–2.4), *p* = 0.019) (Table 4).

**Table 4:** Crude and adjusted hospital, short-term, and long-term outcomes in acute heart failure with concomitant ACS

Covariate	Crude OR (95 % CI)	*P* value	Adjusted OR (95 % CI)	*P* value
Recurrent heart failure	1.24 (1.03–1.48)	0.022	1.66 (1.33–2.06)	<0.001
Overall shock	1.95 (1.47–2.57)	<0.001	1.85 (1.31–2.60)	<0.001
Stroke/TIA	2.44 (1.37–4.32)	0.002	1.87 (0.98–3.56)	0.055
All-cause mortality				
Hospital	1.62 (1.17–2.24)	0.003	1.61 (1.08–2.39)	0.019
1-month	1.40 (1.04–1.89)	0.026	1.36 (0.9–1.95)	0.093
1-year	1.07 (0.86–1.32)	0.548	1.16 (0.89–1.51)	0.252
2-year	1.00 (0.81–1.22)	1.000	1.10 (0.85–1.40)	0.462
3-year	0.98 (0.80–1.20)	0.871	1.07 (0.83–1.35)	0.600

*Abbreviations*: *TIA* transient ischemic attacks

**Fig. 1:**
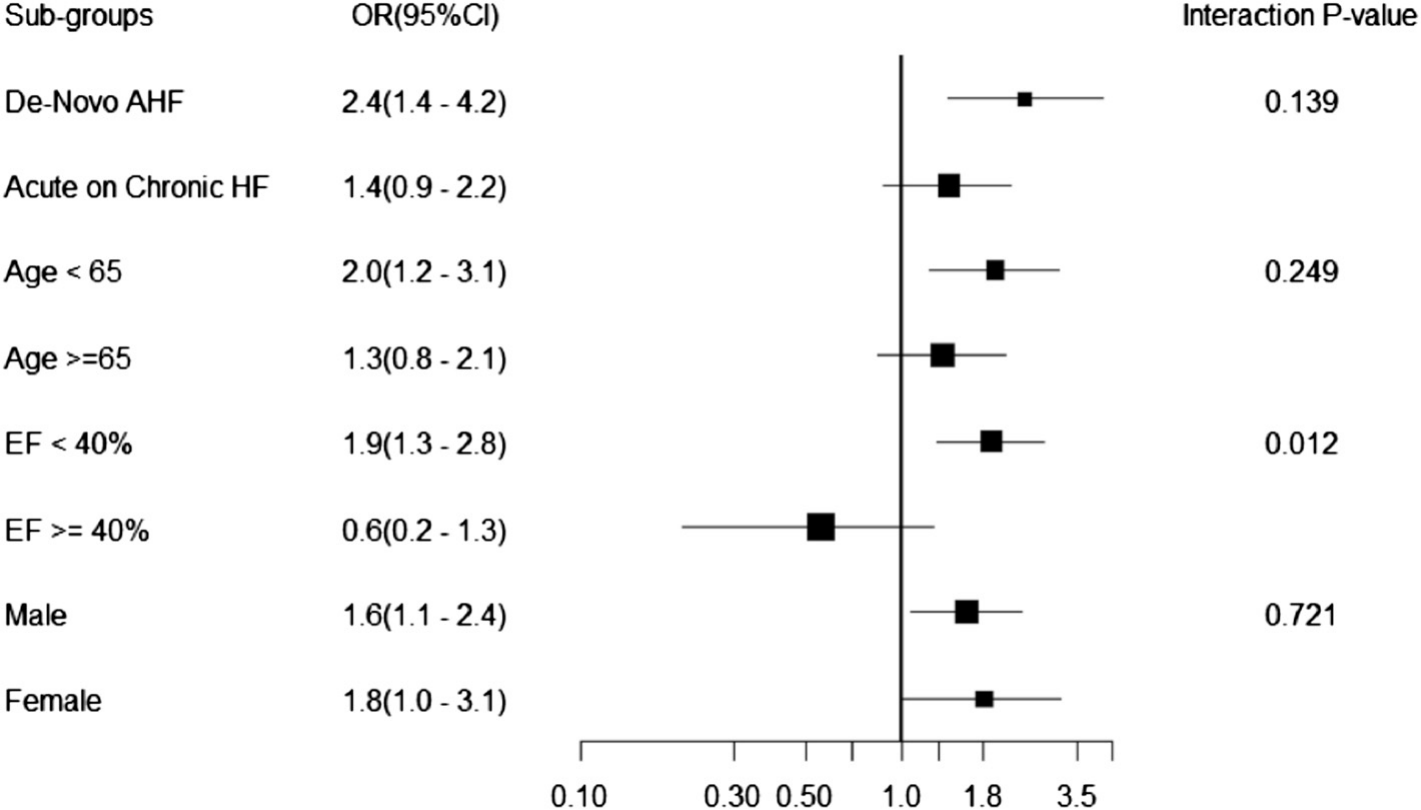
Interaction between acute coronary syndrome with acute heart failure and hospital mortality in selected patients groups

We looked at the interaction between ACS that was concomitant with AHF and hospital mortality in several important clinical groups, including AHF type (de novo and acute on chronic), age groups (≥ or <65 years of age), and LVEF level (≥ or <40 %). AHF with ACS was a predictor of hospital mortality across all selected groups; however, its predictive power was heterogeneous, depending on the LVEF cut-off (for EF < 40 %, OR = 1.9 (95 % CI, 1.3–28) and for EF ≥ 40 %, OR = 0.6 (95 % CI 0.2–1.3), *p* = 0.02 for the interaction) (Fig. 1).

**Fig. 2:**
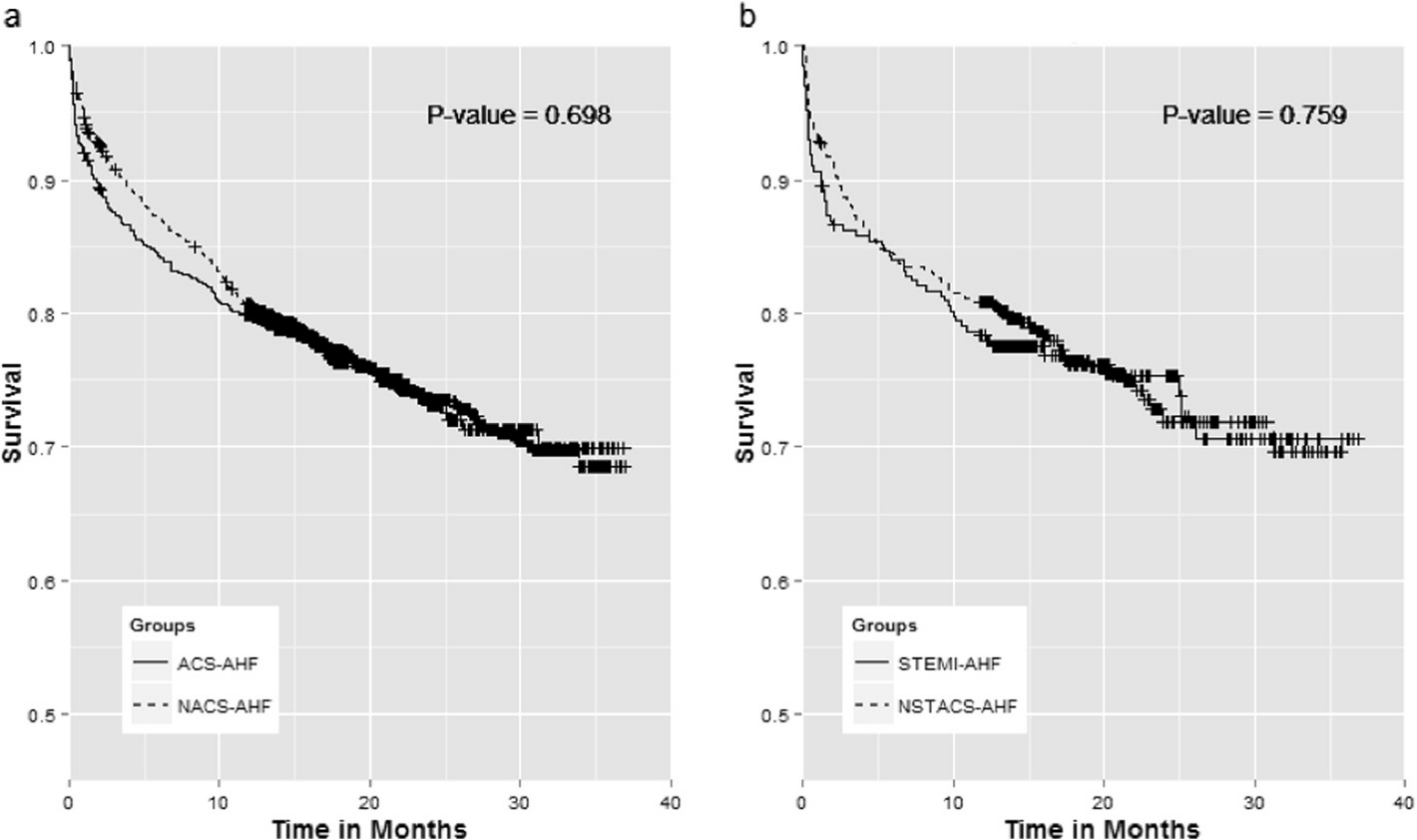
Kaplan-Meier survival curves between: **a** acute heart failure with (*solid line*) and without acute coronary syndrome (*dashed line*), **b** ST elevation myocardial infarction (*solid line*) and Non ST acute coronary syndrome (*dashed line*)

**Fig. 3:**
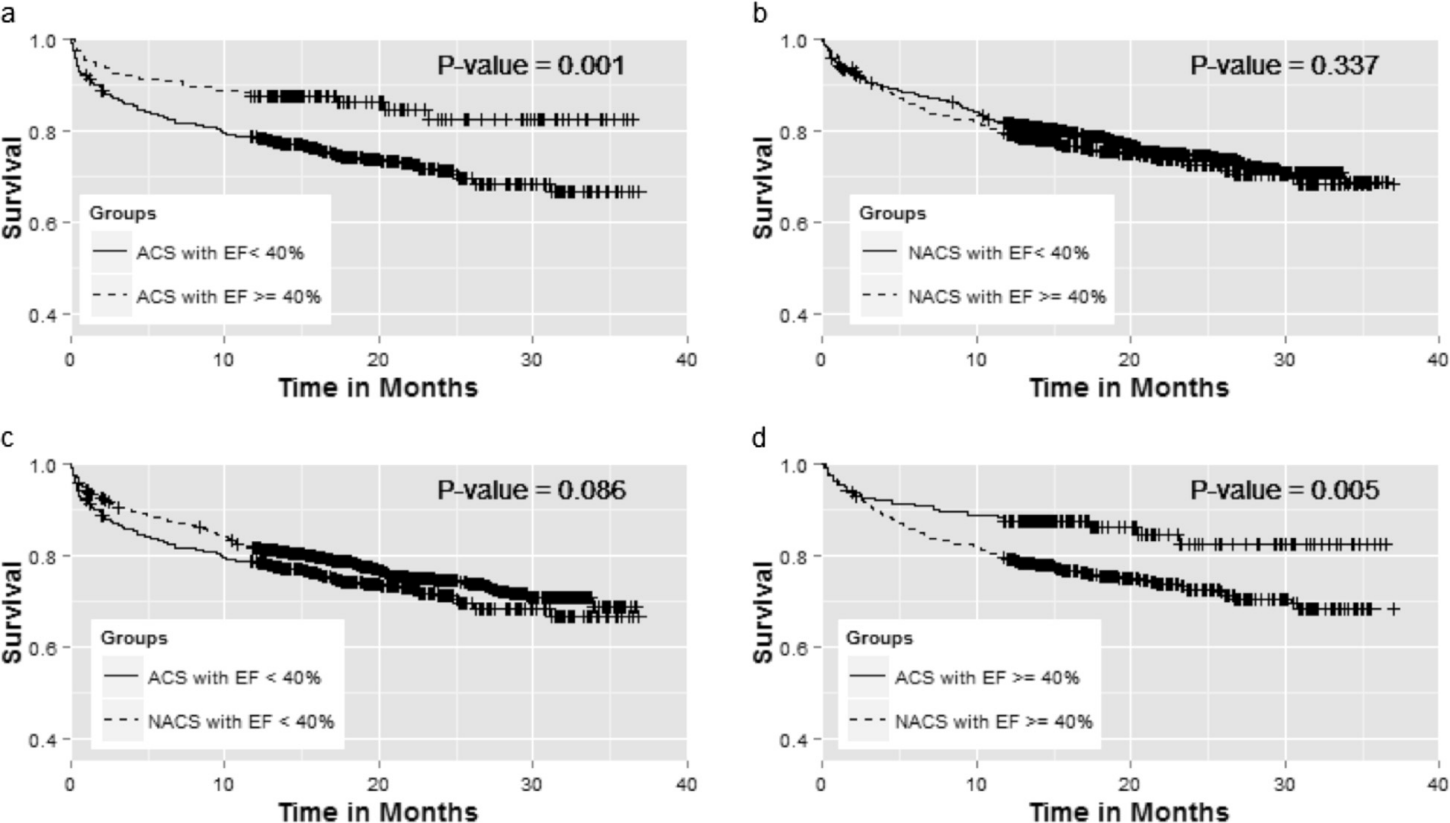
Kaplan-Meier survival curves between: **a** Acute coronary syndrome (ACS) with ejection (EF) <40 % (*solid line*) and ACS with EF ≥ 40 %, **b** No ACS <40 % (*solid line*) and No ACS with EF ≥ 40 %, **c** ACS with EF <40 % (*solid line*) and no ACS with EF <40 % (*dashed line*), **d** ACS with ≥ 40 % (*solid line*) and No ACS with EF ≥ 40 % (*dashed line*)

The 3-year survival of ACS-AHF and NACS-AHF patients was not significantly different (log-rank test, *p* = 0.67) (Fig. 2a). There was no survival difference between AHF patients presenting with STEMI or NSACS (Fig. 2b). The survival of patients with ACS-AHF and LVEF <40 % was significantly lower than that of patients with LVEF >40 % (log-rank test, *p* = 0.001); conversely, no survival difference was noted in patients with NACS-AHF with EF above or below 40 % (Fig. 3a and b). Although an EF < 40 % seemed to have a similar impact on survival in the two groups, ACS-AHF patients had significantly higher survival if the EF was ≥40 % compared to NACS-AHF patients with the same EF cut-off (log-rank test, *p* = 0.005) (Fig. 3c and d).

Because it was clear that the two heart failure groups (ACS-AHF and NACS-AHF) were different, we sought to determine whether the two groups have similar or different predictors of long-term mortality. Therefore, we used logistic regression analysis to identify the 3-year independent predictors of mortality in the two groups. The independent predictors of mortality in ACS patients were age, eGFR, heart rate, and LV systolic function. For patients with no ACS, the independent predictors of mortality were age, DM, hypertension, history of stroke/transient ischemic attacks, RBS, systolic blood pressure, serum urea, and hemoglobin (Table 5).

**Table 5:** Predictors of 3-year all-cause mortality, after adjustment for multiple variates, in acute heart failure with or without acute coronary syndrome

	Odds ratio	95 % CI Lower bound	95 % CI Upper bound	*P* value
Acute heart failure with ACS				
Age (for every 1 year increase)	1.02	1.00	1.04	0.015
eGFR (for every 5 mL/min/1.73 m^2^ decrease)	1.09	1.03	1.16	<0.001
HR (for every 5 beats/min increase)	1.09	1.03	1.15	0.004
Mild LV dysfunction EF 40 %–49.9 %	1.02	0.36	2.87	0.976
Moderate LV dysfunction EF 30–39.9 %	2.37	0.96	5.82	0.060
Severe LV dysfunction EF <30 %	2.78	1.14	6.78	0.024
Acute heart failure without ACS				
Age (for every 1 year increase)	1.03	1.02	1.04	<0.001
Diabetes mellitus	0.61	0.46	0.83	0.001
Hypertension	1.45	1.04	2.01	0.027
Stroke/TIA	1.54	1.06	2.25	0.025
RBS (for every 1 mmol/L increase)	1.03	1.01	1.06	0.006
SBP (for 1 mmHg increase)	0.99	0.99	1.00	<0.001
Urea (for every 1 μmol/L increase)	1.05	1.03	1.06	<0.001
Hemoglobin (for every 1 g/dL increase)	0.88	0.83	0.93	<0.001

*Abbreviations*: *ACS* acute coronary syndrome, *EF* ejection fraction, *eGFR* estimated glomerular filtration rate, *LV* left ventricular, *RBS* random blood sugar, *SBP* systolic blood pressure, *TIA* transient ischemic attacks

## Discussion

Our study is one of a few that compare the outcomes of patients admitted to the hospital with AHF and concomitant ACS to patients with AHF and no ACS in the context of a contemporary HF registry. Approximately a third of the registry population that was admitted with AHF had concomitant ACS, which is in accordance with several previous reports [8, 19–21]. We found that AHF with ACS is a distinct entity with respect to clinical presentation, clinical correlates, and hospital outcomes. ACS-AHF patients were older than NACS-AHF patients and were at higher risk of cardiovascular events by virtue of their past vascular history and risk factors, notably the astonishingly high DM rates. However, they had far fewer comorbidities and were more likely to be HF naïve. This is in stark contrast to the NACS-AHF group, in which more than 70 % had chronic HF.

The main finding of our study was that ACS-AHF patients had higher hospital mortality as well as higher hospital adverse cardiovascular outcomes. Notably, the intermediate and long-term mortality was not different compared to NACS-AHF patients. To our knowledge, only two published reports have addressed the outcomes of a comparable cohort. Our findings are in accordance with the findings of the Finnish Acute Heart Failure Study (FINN-AKVA) with respect to a higher risk of mortality in the short-term and an equivalent risk on the long-term but stand apart from another report that found that the long –term survival is lower in ACS-AHF patients [22, 23]. A few reports have highlighted the unfavorable long-term outcome of acute on chronic HF compared to de novo HF, including a report from our group [24–27]. Yet patients with ACS-AHF in our study, the majority of whom had de novo AHF, were not only at higher risk for hospital adverse cardiovascular outcomes but also had similar long-term survival rates compared to patients with NACS-AHF who predominantly presented with acute on chronic HF. The equivalent long-term survival between the two groups is a somber reality and underscores the excessive risk of mortality beyond hospital discharge and up to three years, presumably because of the older age of the ACS-AHF group, and the extensive coronary artery disease documented in their diagnostic CAG.

ACS concomitant with AHF is an independent predictor of mortality, and its detrimental effect appeared to be consistent across several selected patient subgroups. However, this effect was heterogeneous, depending on the LVEF cut-off that was used (Fig. 1). We found that a low EF adversely impacted survival to a similar degree in AHF patients with and without ACS. Although many studies have demonstrated that HF with reduced EF (HFREF) have higher mortality compared to HF with preserved systolic function (HFPEF) [28], in our cohort this was true only in AHF patients with ACS, presumably because HFPEF patients without ACS in our patient cohort were at very high risk of mortality. Another intriguing finding is that HFPEF patients with no ACS showed significantly lower survival compared to their counterparts with ACS. The reason for the lower survival of HFPEF patients without ACS is unclear, but it is plausible that the lower survival can be explained by the higher prevalence of both cardiac and non-cardiac comorbidities compared to patients with ACS (data not shown). The prognosis of HFPEF patients is strongly linked to underlying non-cardiac comorbidities, such as chronic lung or liver disease [29, 30]. Additionally, anti-HF therapies can often be countered or blunted by pharmacological agents used for treating these non-cardiac comorbidities [29, 30].

Our data highlight several important differences in hospital therapies in the two groups. The high use of IABPs and inotropes in ACS-AHF patients likely reflects the high rates of shock, though it is important to note that the rate of cardiogenic shock was not different between the two groups, contrary to a previous report [22]. The use of nitroglycerine, aspirin, statins, and β-blockers in patients with ACS-AHF is understandably high because these are standard therapies for ACS and IHD in general. The lower use of ACE inhibitors in the NACS-AHF patients might be related to the significantly higher proportion of preserved EF in this group. Additionally, the use of diuretics and AAs was higher in the NACS-AHF patients. Congestive symptoms tend to be higher in patients with chronic HF, and as previously noted, acute on chronic HF was more frequent in the NACS-AHF group [22, 23, 25]. In addition, almost half the NACS-AHF cohort had severe LV systolic dysfunction, and that, along with chronic HF, could explain the higher use of AAs in NACS-AHF patients as well as the higher implantation rates of internal cardiac defibrillators/cardiac resynchronization therapy. Given the fact that the NACS-AHF group more often had a past history of AF, as well more often had incident AF, it is not surprising that the prescription of oral anticoagulation therapy was also high. Overall, the rate of performance of CAG was low in this cohort, and it was performed in less than half of the ACS-AHF cohort. Nonetheless, CAG was performed more than twice as often in patients with ACS-AHF than with NACS-AHF, and the finding of higher significant coronary disease in this group is to be expected. Although we did not collect data on revascularization rates, it is conceivable that these rates are even lower in the context of the low rates of diagnostic CAG. Moreover, 60 % of patients presenting with ACS in our cohort had a past history of IHD; therefore, it could be argued that prior knowledge of their coronary anatomy might have influenced the decision to perform CAG and subsequent revascularization if indicated. Furthermore, some physicians might have scheduled the CAG after hospital discharge when the HF symptoms would be fully resolved.

The reasons underlying the dire outlook of ACS-AHF patients are unclear. Favorable clinical outcomes in ACS depend on rapid diagnosis and risk assessment that leads to timely therapy [17, 31]. The presentation of AHF might on certain occasions obscure concomitant ACS, which in turn could delay risk stratification and rapid treatment [32]. Unfortunately, patients with a combination of ACS and AHF tend to be undertreated and thus to undergo less invasive therapies [11]. Moreover, ischemia often leads to increased myocardial stress and cardiomyocyte damage, and NT-proBNP and troponin are two biomarkers linked to myocardial stress and damage [32]. Indeed, we found that both markers were significantly higher in the ACS-AHF group than in the NACS-AHF group.

Our findings highlight the fact that the intersection of ACS and AHF results in a unique HF entity both in terms of pathophysiology and therapeutic targets. Accordingly, future clinical trials that investigate new therapeutic agents for HF should take into account the uniqueness of this entity rather than including these groups of patients in the general pool of AHF patients.

This study has several limitations. Participation in this registry was voluntary, so selection bias cannot be excluded. Nonetheless, enrolling consecutive patients should minimize this limitation. Although the diagnosis of ACS was guided by the protocol, the diagnosis was based largely on the physician’s judgment of the clinical context and presenting symptoms along with the ECG and cardiac biomarker findings. This could lead to under or over diagnosis of ACS. However, the rates of ACS in our cohort were comparable to those in previous HF registries [8, 19–21]. We did not collect data on hospital coronary revascularization, but the rate is likely to be low in view of the low performance of CAG. We also did not collect post-discharge data pertaining to re-hospitalization, compliance with medical therapy, or revascularization procedures, and these data could influence the long-term outcomes.

## Conclusion

ACS-AHF patients differ from NACS-AHF patients in their baseline characteristics, baseline risk level, and cardiovascular outcomes. Future research should focus on developing timely diagnostic and therapeutic strategies to improve the outcomes of this high-risk group.

## Abbreviations

ACS: acute coronary syndrome
AF: atrial fibrillation
AHF: acute heart failure
CABG: coronary artery bypass grafting
CAG: coronary angiography
CKD-EPI: chronic kidney disease epidemiology collaboration equation
DM: diabetes mellitus
EF: ejection fraction
eGFR: estimated glomerular filtration rate
IABP: intraaortic balloon pump
IHD: ischemic heart disease
NSACS: non-ST elevation acute coronary syndrome
NT-proBNP: N-terminal pro brain natriuretic peptide
OR: odds ratios
PCI: percutaneous intervention
SD: standard deviation
STEMI: ST elevation myocardial infarction
TIA: transient ischemic attack
